# Multi-omics insights into the metabolic reprogramming of host cells triggered by *Entamoeba histolytica* Gal/GalNAc lectin intermediate subunit

**DOI:** 10.1128/spectrum.00381-25

**Published:** 2025-08-20

**Authors:** Yanqing Zhao, Hongze Zhang, Shaokun Pan, Ruixue Zhou, Hiroshi Tachibana, Meng Feng, Xunjia Cheng

**Affiliations:** 1 Department of Medical Microbiology and Parasitology, School of Basic Medical Sciences, Fudan University 600595 , Shanghai, China; 2 Shanghai Institute of Infectious Disease and Biosecurity, Fudan University 12478, Shanghai, China; 3 Department of Infectious Diseases, Tokai University School of Medicine 314462, Isehara, Kanagawa, Japan; University of Huddersfield, Huddersfield, United Kingdom

**Keywords:** *Entamoeba histolytica*, Gal/GalNAc lectin, targeted metabolomics, single-cell transcriptomics, metabolic reprogramming, autophagy

## Abstract

The Gal/GalNAc lectin in ‌*Entamoeba histolytica*‌ is pivotal for its pathogenicity, and its intermediate subunit (Igl) is a crucial virulence factor. Here, the interference of eukaryotically expressed Igl on normal metabolic processes within Caco-2 cells was explored. Targeted metabolomic analysis detected 232 metabolites in Caco-2 cells, revealing 63 with significant alterations after Igl treatment. Notably, the lactate content significantly increased, indicating that Igl activates aerobic glycolysis. Metabolic flux analysis demonstrated that Igl could inhibit aerobic respiration and the tricarboxylic acid cycle in host cells. It significantly increased glucose intake, lactate production, and ATP generation, demonstrating its ability to induce a Warburg-like effect. Through single-cell transcriptomic experiments, significant increases in changes were observed in 4 out of 12 individual host cell groups after Igl treatment. Group marker genes identified these cell groups as having inhibited aerobic respiration and activated stress responses, further indicating that Igl stimulation can affect the aerobic respiration and normal metabolic processes of cells. Additionally, RNA interference experiments were performed to verify the effect of Igl on host cell autophagy when *PRKAA1* was silenced. Confocal microscopy and western blotting revealed significantly enhanced autophagy signals after Igl treatment, and it was confirmed that mammalian target of rapamycin was involved in this process. These results indicate that Igl induces metabolic reprogramming and a Warburg-like shift in host epithelial cells, thereby activating aerobic glycolysis and regulating host cell autophagy. This represents a novel mechanism of Igl in the pathogenicity of *E. histolytica*.

**IMPORTANCE:**

*Entamoeba histolytica* causes amoebiasis, an infection manifesting as colitis and extraintestinal abscesses. Multi-omics approaches provide critical insights into the role of the Gal/GalNAc lectin intermediate subunit (Igl) within host cells, offering a foundation for developing effective treatments. Our findings indicate that Igl induces metabolic reprogramming in host epithelial cells. Specifically, Igl can trigger a Warburg-like shift, a phenomenon characterized by the activation of aerobic glycolysis. This shift results in increased glucose intake, lactate production, and ATP generation while inhibiting aerobic respiration and the tricarboxylic acid cycle. Furthermore, Igl regulates host cell autophagy, a process further confirmed through RNA interference experiments targeting *PRKAA1*, which revealed the involvement of mammalian target of rapamycin. Taken together, our data suggest that Igl promotes trophozoite virulence through a novel mechanism that involves metabolic reprogramming and a Warburg-like shift in host cells.

## INTRODUCTION

*Entamoeba histolytica*, the causative agent of amoebiasis, is estimated to cause 50 million cases of symptomatic disease worldwide, resulting in between 40,000 and 100,000 deaths annually (1–3). Galactose (Gal)-inhibitable and N-acetyl-d-galactosamine (GalNAc)-inhibitable lectin is a major factor in mediating *E. histolytica* adherence, and its intermediate subunit (Igl) is an important surface antigen and virulence factor (4–6). Igl not only mediates amoebic adherence to host cells but also acts as an efficient surface antigen to elicit various immunological responses while producing multiple specific anti-Igl antibodies *in vivo*, some of which can in turn inhibit the binding of *E. histolytica* trophozoites to host cells (7, 8). In terms of pathogenic effects, Igl has significant hemolytic and cytotoxic activities, mainly reflected by its C-terminus (5, 9). Within an *E. histolytica* trophozoite, Igl can also conduct cell signals through protein-protein interactions with elongation factor-1 alpha and transmembrane kinases, jointly regulating amoebic migration and adhesion (10, 11).

The success of *E. histolytica* as a pathogen is due to the elaborate mechanisms it uses to complete each step of its life cycle and evade host cell defenses. Remarkably, this parasite is almost entirely dependent on the nutrient supply in the host environment for survival (12). Thus, parasite-host metabolic interactions are essential for amoebic trophozoites because they require the metabolites of host cells to meet their energy needs. This dependency underpins parasite biosynthesis, the release of receptor complexes that enhance adherence and phagocytosis, and the ability to establish chronic infections resistant to immune responses and antiparasitic drugs (13). Our recent study found that *E. histolytica* trophozoites could induce metabolic reprogramming in host cells during the early stages of infection, presenting significant suppression of central carbon metabolism and gene expression changes in glycolysis and respiratory chain enzymes (14). Moreover, Igl plays an important role in this pathogenic process. The Warburg-like effect induced by *E. histolytica* trophozoites and Igl represents a novel pathogenic mechanism, highlighting the metabolic alterations in host cells during parasite-host interactions.

While cellular metabolism usually involves different metabolites, changes in the levels of various intracellular metabolites during such stimulation have not yet been systematically investigated (15). How Igl influences cell fate through metabolic reprogramming and activates specific cellular signaling pathways remains uncertain. Moreover, our previous quantitative proteomics research was primarily a bulk analysis at the multicellular level and lacked analyses at the single-cell level, which may have yielded new information and mechanisms (14). In light of these requirements, to further validate the specific mechanisms of Igl-induced Warburg-like effect during *E. histolytica* infection, the present study utilized metabolomics, metabolic flux analysis, and single-cell transcriptomics to explore metabolic alterations and ensuing cellular regulation in host intestinal epithelial cells after eukaryote-expressed Igl stimulation.

## RESULTS

### Targeted metabolomics of central carbon metabolism in host Caco-2 cells after Igl stimulation

Central carbon metabolism refers to the process by which cells take up glucose and decompose it to produce ATP through complex biochemical reactions, such as glycolysis, the tricarboxylic acid (TCA) cycle, and oxidative phosphorylation. Based on our initial proteomic research, to further study the metabolic reprogramming of host cells induced by Igl from the perspective of metabolites, we first designed targeted metabolomics for the corresponding substances. For an overview analysis based on quality control (QC) samples, the relative standard deviations (RSDs) of all reported ion peaks were <0.5, whereas the median and mean were both <0.2 (Fig. S1A). In the case of clear stratification among the different groups, the QC samples were well concentrated in principal component analysis (PCA) (Fig. S1B). The RSD of the stable isotope-labeled internal standard was <0.2, indicating that all samples were properly injected during the detection process (Fig. S1C).

**Fig 1:**
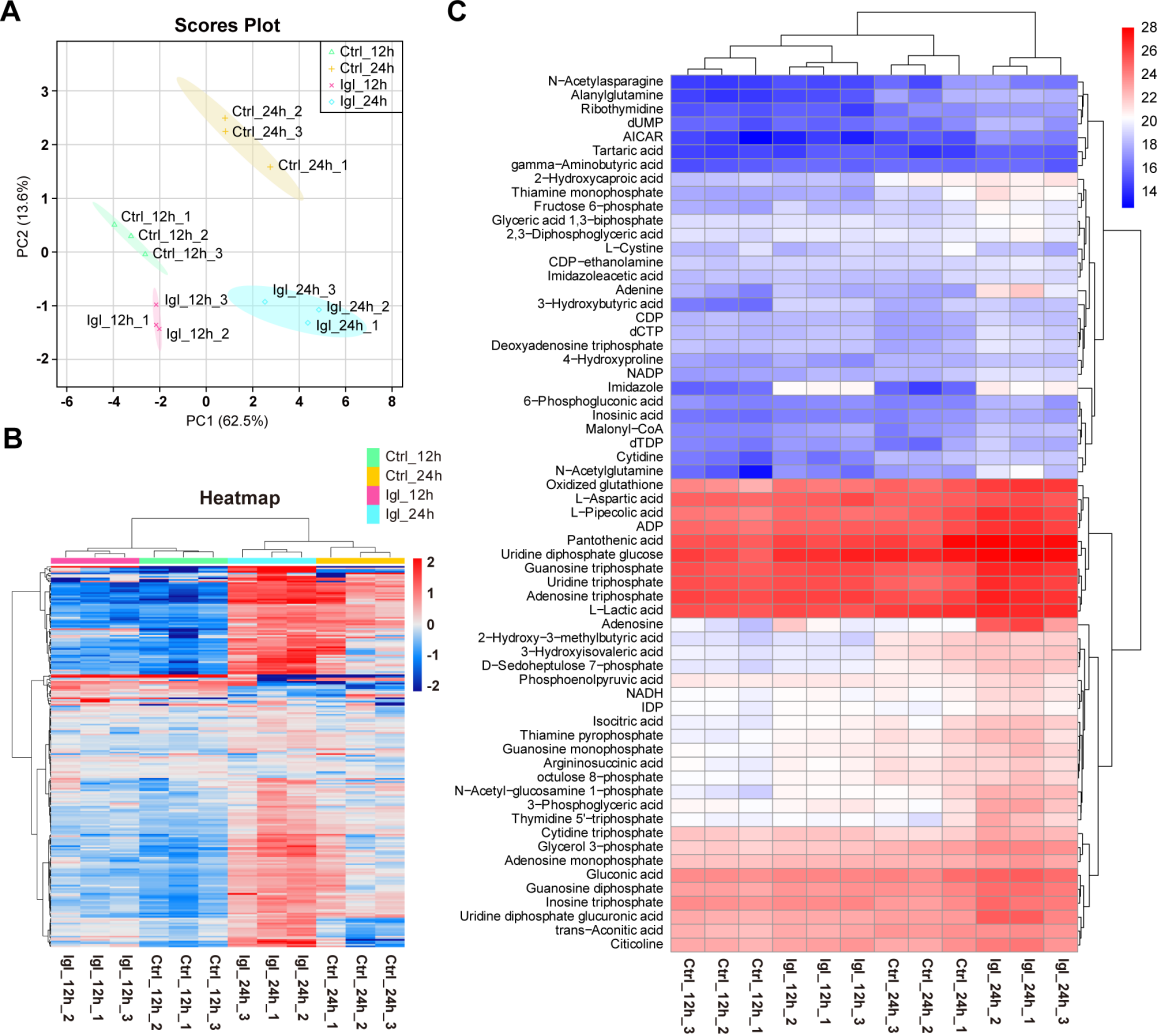
Principal component analysis and heat maps of metabolite changes in targeted metabolomics. (**A**) Principal component analysis of all control and Igl-treated groups. (**B**) Overall heat map of all detected metabolites. (**C**) Heat map of significantly upregulated or downregulated metabolites in Caco-2 cells after Igl stimulation.

As shown in the PCA, the differences within each group were small, whereas those between groups were significant (Fig. 1A). With persistent Igl stimulation, changes in central carbon metabolism in Caco-2 cells became more significant. The 232 detected metabolites were mapped onto a global heat map to reflect the overall changes and clustering of metabolites in Caco-2 cells, where each column represents a sample and each row represents a metabolite ion peak (Fig. 1B). Using log2FC (fold change) > 0.4 or log2FC < −0.4 and false discovery rate (FDR) value < 0.16 (Student’s *t*-test with multiple hypothesis testing correction) as the criteria to determine the significance of changes, 63 intracellular metabolites changed markedly after Igl stimulation for 12 or 24 h, accounting for 27.16% of all metabolites detected in the cells (Fig. 1C). The significantly altered metabolites were mainly related to cellular energy supply and redox homeostasis and involved various amino acids, suggesting that *E. histolytica* Igl stimulation could affect the central carbon and amino acid metabolism of host cells.

**Fig 2:**
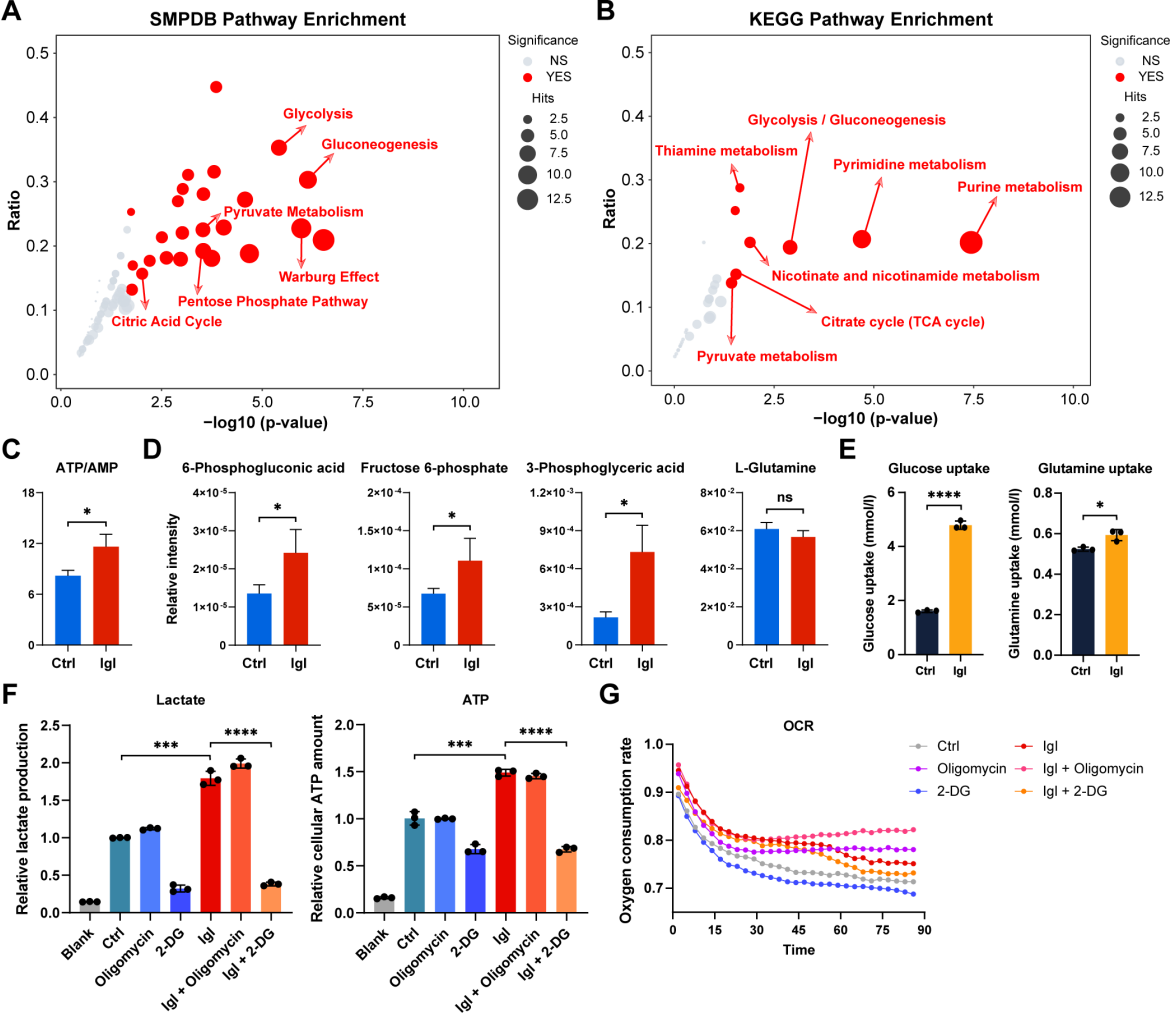
Igl induces metabolite changes of the Warburg-like effect in host Caco-2 cells. (**A**) SMPDB enrichment analysis of differentially regulated metabolites after Igl treatment. (**B**) KEGG enrichment analysis of differentially regulated metabolites after Igl treatment. (**C**) Changes in the ratio of cellular ATP to AMP after Igl treatment. (**D**) Changes of metabolites associated with glycolysis and glutaminolysis in host Caco-2 cells, including 6-phosphogluconic acid, fructose 6-phosphate, 3-phosphoglyceric acid, and L-glutamine. (**E**) Changes of glucose uptake and glutamine uptake in host Caco-2 cells after Igl treatment. (**F**) Changes of lactate production and ATP level in Igl-treated Caco-2 cells. Oligomycin and 2-deoxyglucose were separately added to inhibit the oxidative phosphorylation and glycolysis of the cells. (**G**) Variations in oxygen consumption rate of Igl-treated Caco-2 cells under oligomycin or 2-deoxyglucose inhibition. Data are all expressed as mean with standard deviation (SD) (*n* = 3, Student’s *t*-test). *P  <  0.05, ***P  <  0.001, and ****P  <  0.0001.

To explore the functions and pathways of the differentially regulated metabolites, functional annotation and enrichment analysis of differential metabolites were performed using the SMPDB and KEGG (Kyoto Encyclopedia of Genes and Genomes) databases. Setting *P* < 0.05 (with multiple hypothesis testing correction) as the criterion, the metabolic pathways that were significantly regulated after IgG stimulation are shown in bubble charts. SMPDB enrichment analysis showed that the changes in metabolites were concentrated in pathways closely related to aerobic respiration, anaerobic respiration, glucose decomposition, and anabolism (Fig. 2A). Meanwhile, the results of the KEGG enrichment analysis exhibited that Igl stimulation was related not only to central carbon metabolism but also to the nucleotide metabolism of host cells (Fig. 2B).

To further study the specific regulatory mechanism of Igl in host cells, relevant metabolite data detected by targeted metabolomics were applied to generate histograms (Fig. 2C). After Igl stimulation for 24 h, the ratio of cellular ATP to AMP significantly increased, indicating an enhancement in energy supply. The levels of 6-phosphogluconic acid, fructose 6-phosphate, and 3-phosphoglyceric acid (the intermediate molecules of glycolysis) were significantly increased, while the level of L-glutamine (the substrate for glutaminolysis) in Caco-2 cells remained unchanged, indicating that the enhancement in energy supply occurred through the heightening of glycolysis, and Igl had little effect on glutaminolysis (Fig. 2D). Subsequently, quantitative detection kits for metabolites were used for validation (Fig. 2E). Caco-2 cells exhibited a significant increase in glucose uptake, while that of glutamine only had a slight change. We separately added oligomycin and 2-deoxyglucose to inhibit the oxidative phosphorylation and glycolysis of the cells, finding that the increases in lactate production and cellular ATP amount caused by Igl treatment could be significantly inhibited by 2-deoxyglucose, while oligomycin was ineffective (Fig. 2F). Finally, variations in oxygen consumption rate of Igl-treated Caco-2 cells under oligomycin or 2-deoxyglucose inhibition were detected for confirmation (Fig. 2G).

Our results showed that *E. histolytica* Igl stimulation could induce metabolic reprogramming in host cells. Through the detection of energy substance uptakes and the use of energy metabolism inhibitors, this Igl-mediated metabolic reprogramming was confirmed to be mainly achieved through an enhancement of glycolysis and a transition from aerobic respiration to anaerobic respiration. During this process, only the central carbon metabolism significantly shifted, and glutaminolysis was not found to be directly affected. Owing to the lowered efficiency of glucose decomposition to produce ATP, Caco-2 cells took up more glucose and produced more lactate to maintain their energy supply, which is similar to the process of aerobic glycolysis in tumor cells (the Warburg effect).

### Metabolic flux analysis revealed correlation of the Warburg-like effect in host Caco-2 cells with Igl

**Fig 3:**
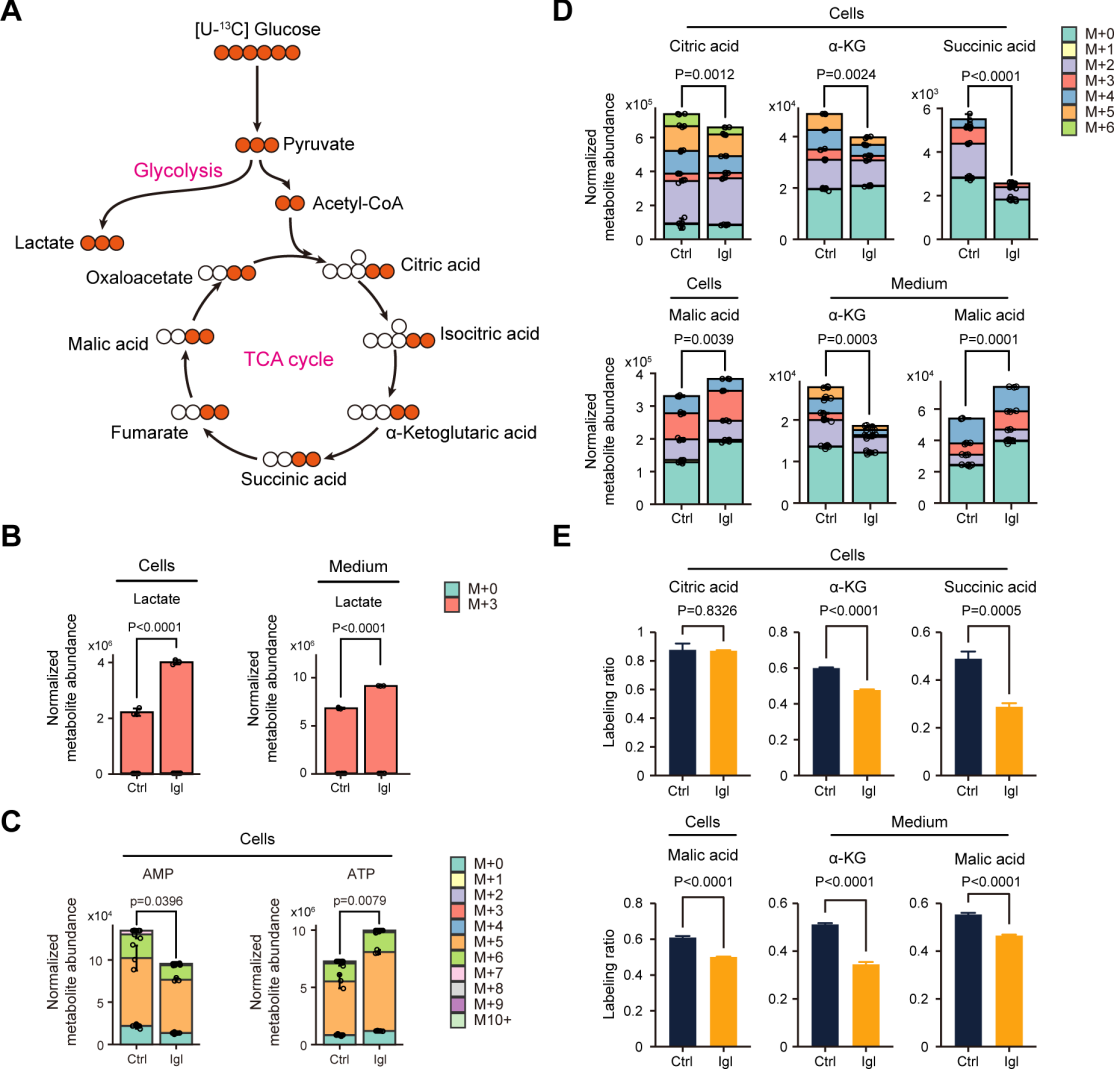
Detection of metabolic flux in Caco-2 cells after Igl stimulation. (**A**) Schematic diagram of central carbon metabolism in metabolic flux analysis. (**B**) Bar charts displaying the lactate levels in Caco-2 cells and culture medium. (**C**) AMP and ATP levels in Caco-2 cells detected by metabolic flux analysis. (**D**) Contents of total and isotopomer of multiple intermediates in the TCA cycle, including citric acid, α-ketoglutaric acid, succinic acid, and malic acid. (**E**) Percentage of isotopomer for the TCA circulation intermediates, including citric acid, α-ketoglutaric acid, succinic acid, and malic acid. Data are all expressed as mean with SD (*n* = 3, Student’s *t*-test).

In the normal aerobic respiration of cells, glucose is decomposed into pyruvate, which is then converted into acetyl-CoA and enters the TCA cycle, producing abundant ATP. However, when the cellular oxygen supply is insufficient, anaerobic respiration is mainly used to supply energy, and pyruvate is eventually converted to lactate. In this study, glucose in the Caco-2 cell medium was completely replaced with [^13^C_6_]-glucose, followed by *E. histolytica* Igl addition to the experimental group wells for 24 h (Fig. 3A). Under the influence of Igl, the lactate content in both Caco-2 cells and culture supernatants was significantly upregulated, suggesting that the aerobic glycolysis of host cells was activated (Fig. 3B). Meanwhile, the proportion of carbon replaced by ^13^C (annotated as M + 3) in lactate exceeded 99%, indicating that the isotope-labeled cell metabolism had already stabilized.

Activation of aerobic glycolysis leads to an increased conversion of pyruvate to lactate, which may result in aerobic respiration inhibition and TCA cycle blockage. After *E. histolytica* Igl stimulation, total AMP was significantly downregulated, and total ATP was significantly upregulated in Caco-2 cells (Fig. 3C). Analysis of TCA cycle intermediates revealed that Igl treatment significantly decreased citric acid, α-ketoglutaric acid (α-KG), and succinic acid levels, while malic acid levels were significantly elevated (Fig. 3D). Similarly, in culture supernatants, α-ketoglutaric acid content decreased, and malic acid content increased after Igl stimulation.

To further investigate the variation trends of aerobic respiration and the TCA cycle, the percentage of isotopomers among these key intermediates (the sum of each metabolite detected, except M + 0) was analyzed. As shown in Fig. 3E, the isotopomers of the above metabolites were significantly downregulated in both Caco-2 cells and culture supernatants, suggesting that Igl treatment inhibited the TCA cycle pathway. Notably, the percentage of malic acid isotopomers detected in the cells and supernatants decreased, whereas the total malic acid content increased. This result showed that the consumption of malic acid was inhibited; that is, the production of oxaloacetic acid from malic acid in the TCA cycle was inhibited by Igl.

### Igl-dependent metabolic reprogramming in host Caco-2 cells verified at the single-cell level

Using Cell Ranger version 7.1.0, data QC was conducted to filter low-quality information, such as fragmented, double, and dead cells, after single-cell sequencing (Fig. S2A). The filtering criteria were set as follows: 200 < total expressed gene number < 10,000, transcript number > 500, and mitochondrial gene content < 10%. This process yielded 9,069 and 8,713 single cells from control (Ctrl) and experimental (Igl) samples, respectively, for subsequent analysis. Single cells from the control and Igl-treated samples were clustered and visualized, as shown in Fig. S2B. In the integrated analysis of all single-cell transcriptional data, 10,045 genes were detected, among which log2FC > 0.25 or log2FC < –0.25 and *P* < 0.05 (Student’s *t*-test with *P* value corrected by multiple hypothesis testing) were set as the criteria to determine the significance of gene expression changes. A volcano plot was constructed with the relative difference in gene expression as the horizontal coordinate and log2FC as the vertical coordinate, where red dots represent genes with significantly upregulated expression and green dots represent genes with significantly downregulated expression after Igl stimulation (Fig. S2C). Compared to the control group, the expression of 558 genes was significantly altered, including 266 upregulated and 292 downregulated ones.

**Fig 4:**
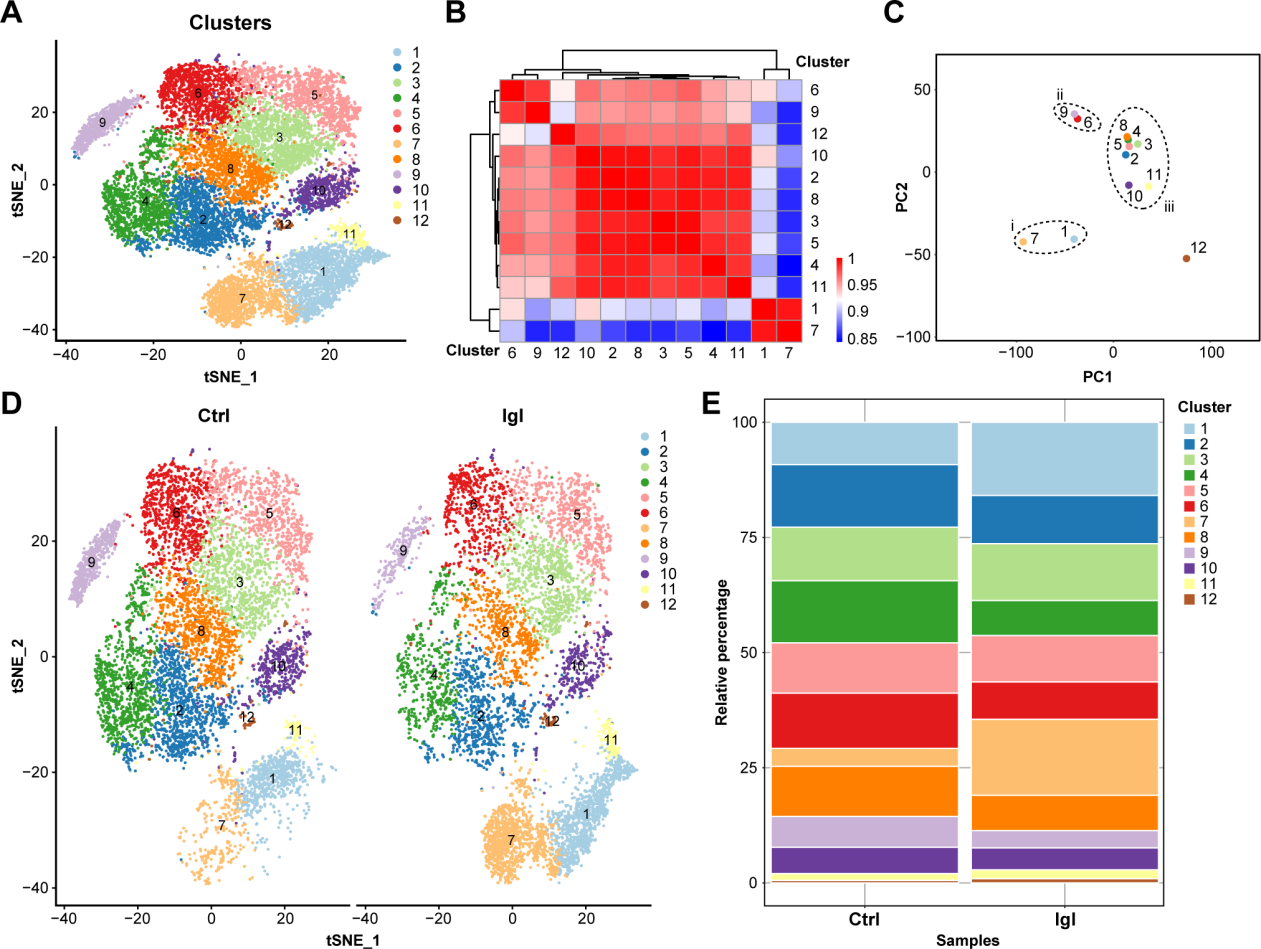
Effects of Igl stimulation on transcriptional profiles of Caco-2 cell clusters. (**A**) Caco-2 cell clustering by *t*-distributed stochastic neighbor embedding (*t*-SNE) analysis. (**B**) Correlation heat map of all cell clusters. (**C**) Principal component analysis of all cell clusters. (**D**) Clustering differences in Caco-2 cells after Igl treatment. (**E**) Cell population change in each cluster after Igl treatment.

Owing to cell heterogeneity, a few cells with significant changes during Igl stimulation may be masked by most cells. Thus, in the present single-cell analysis, Caco-2 cells were clustered, and the marker genes of each cluster were identified for in-depth comparison. A total of 17,782 cells from both the control and experimental groups were integrated and clustered to obtain 12 different clusters in various cell states (Fig. 4A). As shown in the correlation heat map, strong correlations existed between clusters 1 and 7, clusters 6 and 9, and clusters 2, 3, 4, 5, 8, 10, and 11 (Fig. 4B). In the PCA, clusters with strong correlations were close to each other, whereas those with weak correlations were clearly separated (Fig. 4C). The effects of Igl stimulation on each cell cluster are shown in Fig. 4D. Subsequently, the relative cell number of each cluster in control and experimental groups was counted and chi-square tested, exhibiting a significant increase in cell numbers of clusters 1 (*P* < 0.0001), 7 (*P* < 0.0001), 11 (*P* < 0.05), and 12 (*P* < 0.01), and a significant decrease in cell numbers of clusters 2 (*P* < 0.0001), 4 (*P* < 0.0001), 6 (*P* < 0.0001), 8 (*P* < 0.0001), 9 (*P* < 0.0001), and 10 (*P* < 0.01) (Fig. 4E).

### Igl-induced energy metabolism reprogramming in Caco-2 cells analyzed through single-cell transcriptomics

**Fig 5:**
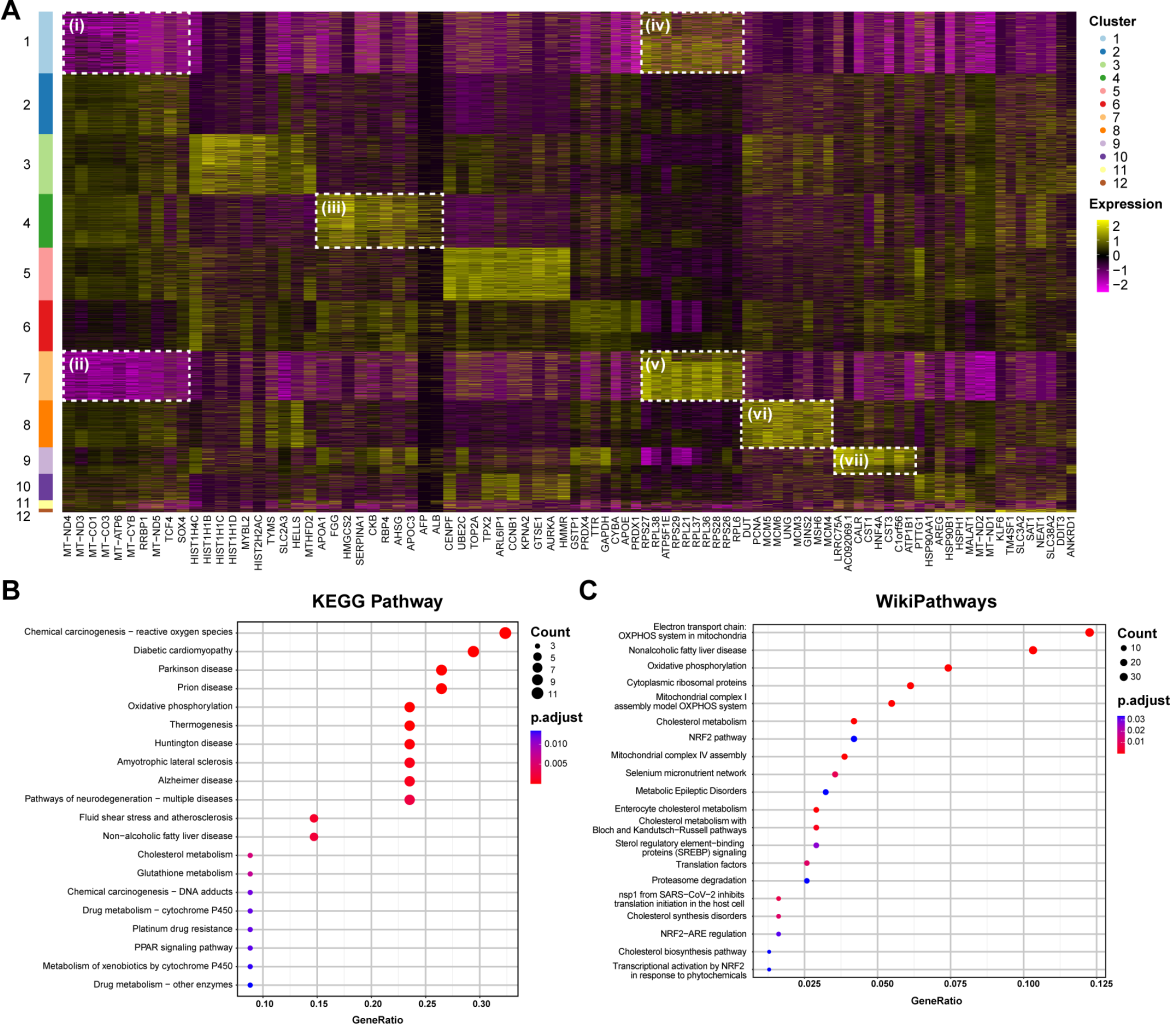
Various clustering analyses in single-cell RNA-seq transcriptomics. (**A**) Enrichment heat map of the top 10 marked genes of each Caco-2 cell cluster. (**B**) Top 20 pathways with highest enrichment levels in the KEGG pathway enrichment analysis of differentially expressed genes. (**C**) Top 20 pathways with highest enrichment levels in the WikiPathways enrichment analysis of differentially expressed genes.

Using the FindAllMarkers function in Seurat, the marker genes of each cell cluster were further screened, and the top 10 most significantly different genes were selected for visual heat map enrichment (Fig. 5A), with *P* < 0.05 and FC ≥ 1.5 set as the criteria. Compared to other clusters, the transcription levels of mitochondria-related genes, such as NADH-ubiquinone oxidoreductase chain genes, cytochrome C oxidase genes, and ATP synthase genes, in clusters 1 and 7 were significantly downregulated (Fig. 5A, panels i and ii). These genes are all involved in respiration and energy supply, and their reduction suggests that cellular respiration was inhibited in clusters 1 and 7. In contrast, the transcript levels of genes related to mitochondrial respiration and ATP synthesis in cluster 2 were higher than those in the other clusters. After Igl stimulation, the proportion of cells in clusters 1 and 7 increased, and the proportion of cells in cluster 2 decreased, indicating that Igl generally inhibited cellular respiration and normal energy supply. In addition, the transcription levels of ribosome-related and transcription-related genes were significantly higher in clusters 1 and 7, suggesting that Igl stimulation may affect the normal transcription and translation processes in Caco-2 cells (Fig. 5A, panels iv and v).

After Igl treatment, the cell proportions of clusters 11 and 12 also increased, and the expression of their transcription factor KLF6, transmembrane protein TM4SF1, amino acid transporters SLC3A2 and SLC38A2, acetyltransferase SAT1, and DNA damage-inducible transcript DDIT3 was significantly higher, indicating that Igl may activate various transcription factors and enhance the transmembrane transport of amino acids to promote the cell response to external stress. Combined with the reduced cell number in cluster 4 and the function of its highly expressed genes, Igl may inhibit lipid transport, ketogenesis, and endocytosis in host cells (Fig. 5A, panel iii). Ketogenesis is a stress response to insufficient glucose uptake or lack of energy reserves. This suggests that *E. histolytica* Igl can induce metabolic reprogramming and affect the normal energy supply in host cells. Additionally, the results of cluster 8 indicated Igl inhibition of DNA replication, division, and proliferation in host cells, while the results of cluster 9 suggested Igl inhibition of genes related to innate immunity (Fig. 5A, panels vi and vii).

To more intuitively show the relationship between differentially expressed genes (DEGs) and the corresponding molecular pathways induced by Igl, KEGG pathway enrichment analysis and WikiPathways enrichment analysis were performed, in which the top 20 entries with the highest enrichment and the most genes were displayed as dot plots. In the KEGG enrichment results, transcriptional changes induced by Igl were focused on pathways such as oxidative phosphorylation, thermogenesis, cholesterol metabolism, glutathione metabolism, and the peroxisome proliferator-activated receptor signaling pathway (Fig. 5B). The results of WikiPathways enrichment analysis showed that differentially expressed genes in Caco-2 cells were mainly related to the mitochondrial electron transport chain, oxidative phosphorylation, cytoplasmic ribosomal proteins, cholesterol metabolism, proteasome functions, mammalian target of rapamycin (mTOR) signaling pathway, and autophagy, which were generally consistent with those of the KEGG pathway enrichment analysis (Fig. 5C; Table S1).

### Igl regulates energy metabolism and activates autophagy in host Caco-2 cells

**Fig 6:**
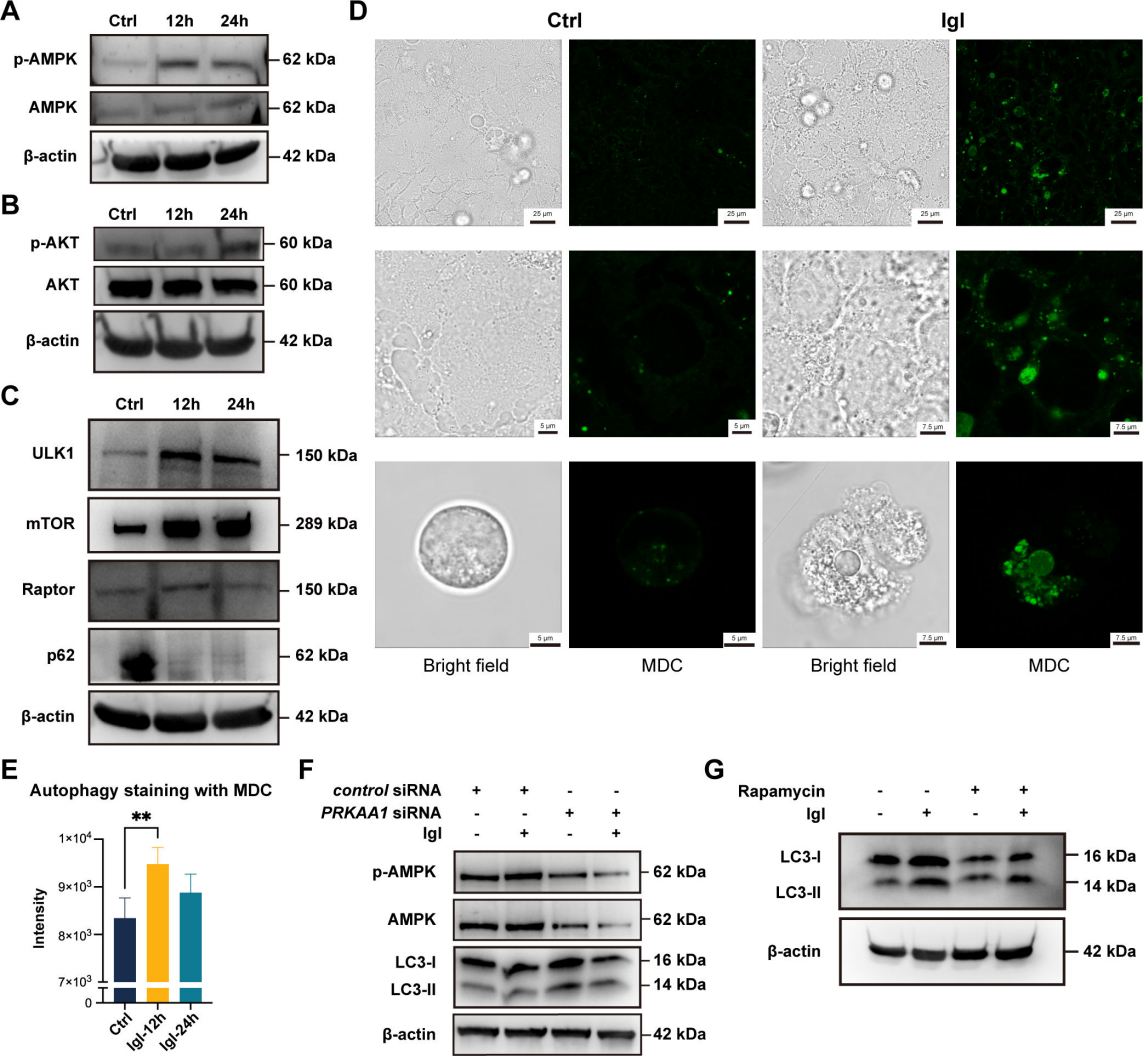
Igl regulates energy metabolism and activates autophagy in host Caco-2 cells. (**A**) Increased AMPK phosphorylation in Caco-2 cells after Igl stimulation. (**B**) Increased AKT phosphorylation in Caco-2 cells after Igl stimulation. (**C**) Western blotting showing changed protein levels of ULK1, mTOR, raptor, and p62 in Caco-2 cells after Igl stimulation. By monodansylcadaverine (MDC) staining, laser confocal microscopy (**D**) and microplate reader quantification (**E**) were performed for autophagy detection of Caco-2 cells after Igl treatment. Data are expressed as mean with SD (*n* = 4, Student’s *t*-test). (**F**) AMPK was involved in host Caco-2 cell autophagy induced by Igl. RNA interference was performed to silence the *PRKAA1* gene. (**G**) mTOR was involved in host Caco-2 cell autophagy induced by Igl. Rapamycin was added for mTOR inhibition. **P  <  0.01.

As a key molecule that regulates energy metabolism, AMPK responds to changes in intracellular ATP levels and regulates metabolic processes such as glucose uptake, glycolysis, fatty acid oxidation, and autophagy through a series of signaling pathways (16). In addition to AMPK, AKT is another important molecule that responds to energy stress and redox stress, which not only promotes cell survival and inhibits apoptosis but also regulates glucose metabolism and glycolysis (17). Western blotting was performed to detect changes in intracellular AMPK and AKT phosphorylation levels after Igl stimulation. As expected, the phosphorylation levels of AMPK and AKT significantly increased, with the former being more significant at 12 h and the latter being more significant at 24 h (Fig. 6A and B).

Phosphorylation of AMPK and AKT can activate corresponding downstream pathways and regulate cell growth and stress, which involve various molecules, including Unc-51-like autophagy-activating kinase 1 (ULK1) and mTOR (16). After Igl stimulation, changes in ULK1, mTOR, raptor, and p62 (an autophagy marker) protein levels in Caco-2 cells were detected (Fig. 6C). The expression of ULK1, mTOR, and raptor significantly increased, whereas that of p62 significantly decreased, suggesting that the parasite could activate autophagy through Igl. Notably, the upregulation of ULK1 and raptor was most significant at 12 h, indicating that host cell autophagy might be more intense in the early stage. In addition to detecting autophagy markers, the autophagy status of cells can be determined by labeling autophagosomes with monodansylcadaverine. Both confocal microscopy and microplate reader quantification revealed significantly enhanced autophagosome signals in the Igl-stimulated group, indicating that Igl activates host cell autophagy (Fig. 6D and E).

RNA interference experiments were designed to verify the effect of *E. histolytica* Igl on host cell autophagy when *PRKAA1* (responsible for AMPK expression) was silenced (Fig. 6F). Microtubule-associated protein 1 light chain 3 (LC3) is another important autophagy marker, and the levels of LC3-I and LC3-II reflect changes in autophagy. After *PRKAA1* silencing, the expression of AMPK, phosphorylated AMPK, LC3-I, and LC3-II proteins stimulated by IgG was significantly inhibited, suggesting that autophagy was inhibited. Rapamycin is an inhibitor of mTOR that mainly promotes autophagy by inhibiting mTOR activity. After treatment with 20 nM rapamycin, LC3-I levels in Caco-2 cells decreased, and LC3-II levels were upregulated relative to LC3-I levels, demonstrating the ability of rapamycin to activate autophagy (Fig. 6G). Igl stimulation increased the expression of both LC3-I and LC3-II in Caco-2 cells, whereas rapamycin pretreatment inhibited the increase in LC3-II protein levels induced by Igl and weakened the upregulation of LC3-II relative to LC3-I, suggesting that mTOR is indeed involved in the regulation of Igl-induced host cell autophagy.

## DISCUSSION

The Warburg effect, a metabolic reprogramming phenomenon characterized by a shift from oxidative phosphorylation to glycolysis, was first described in cancer research (18). Subsequent studies in infectious diseases revealed that pathogens can induce similar metabolic alterations in host cells, promoting a transition from aerobic to anaerobic metabolism. For instance, human adenovirus and hepatitis B virus have been shown to activate host cell glycolysis, modify carbon source utilization, suppress amino acid catabolism, and thereby facilitate viral nutrient acquisition and proliferation (19, 20). While this phenomenon is well documented for intracellular pathogens, our study focuses on *E. histolytica*, an extracellular parasitic protozoan. The Gal/GalNAc lectin intermediate subunit, a major virulence factor of *E. histolytica*, induces host cell metabolic reprogramming—a finding that provides important insights into the invasion mechanisms of extracellular pathogens.

Igl, a membrane protein of *E. histolytica*, mediates parasite adhesion to and phagocytosis of host cells (6–8). Although previous studies have identified host cell membrane receptors for Igl, its additional functions remain poorly characterized. Our targeted proteomics and isotope labeling experiments demonstrate that Igl induces metabolic reprogramming in Caco-2 cells, characterized by enhanced glycolysis and lactate production. This glucose metabolism regulation likely reduces reactive oxygen species (ROS) generation from aerobic metabolism, creating an infection-favorable microenvironment while simultaneously modifying host cell carbon source utilization to support parasite nutrient acquisition and proliferation. These findings suggest a novel pathogenic mechanism employed by *E. histolytica*.

The Warburg effect serves to maintain cellular redox balance and counteract growth-inhibitory factors, including infection-induced oxidative stress, hypoxia, apoptosis, and toxic stimuli (21). Pathogens such as *Salmonella* promote glycolysis through succinate accumulation and HIF-1α upregulation, while *Mycobacterium tuberculosis* enhances lactate synthesis and activates the AMPK/mTOR pathway to induce autophagy (22, 23). Using glycolysis/OXPHOS assays, we observed that Igl-induced lactate production in Caco-2 cells was significantly inhibited by 2-deoxyglucose (2-DG) but unaffected by TCA cycle inhibition. Complementary glucose and glutamine uptake experiments further confirmed the glycolysis-dependent nature of this lactate production. These results indicate that Igl suppresses host cell aerobic metabolism, thereby reducing ROS generation and creating an infection-permissive microenvironment while modulating intracellular carbon flux to support parasite survival.

Igl-induced Warburg-like effects may also stimulate autophagy, a critical process for protein degradation that participates in innate and adaptive immunity, anti-infective responses, and parenchymal cell metabolic reprogramming (24, 25). Metabolic reprogramming in various cell types can trigger lysosomal autophagy through energy-sensing pathways such as AMPK/ULK1/mTOR (26–28), ultimately influencing cell fate (29, 30). Our experiments using gene silencing and pharmacological inhibitors confirmed that Igl-mediated metabolic reprogramming upregulates AMPK and AKT phosphorylation, increases ULK1 and mTOR protein levels, and activates the AMPK/ULK1/mTOR pathway, thereby promoting autophagy in host epithelial cells. This autophagic response represents an adaptive host cell mechanism that maintains intracellular homeostasis, repairs organelle damage (particularly in mitochondria), and initiates inflammatory responses during infection.

Current *in vitro* models for *E. histolytica* infection, primarily using Caco-2 and CHO cells, present limitations for carbon metabolism studies due to their tumorigenic properties. However, our animal model data corroborate these findings, demonstrating Warburg-like effects in the intestinal epithelium of *E. histolytica*-infected C3H/HeNCrl mice (14). Glutamine metabolism assays further confirmed that Igl does not significantly alter glutamine utilization. Despite the inherent limitations of Caco-2 cells, converging evidence from multiple approaches indicates that *E. histolytica* surface protein Igl inhibits host cell aerobic respiration and TCA cycle activity while enhancing glycolysis. As model systems are crucial for metabolic reprogramming studies, future work will employ organoid or co-culture models to improve experimental accuracy.

In summary, Igl mediates significant alterations in host cell central carbon metabolism, creating an infection-favorable microenvironment through ROS reduction and carbon source redistribution. Concurrent host cell adaptive responses, including autophagy, serve to maintain intracellular homeostasis, repair organelle damage, and modulate inflammation. Our findings reveal a novel Igl-dependent mechanism in *E. histolytica* pathogenesis, demonstrating the parasite’s active manipulation of host metabolism to establish infection. This work significantly advances our understanding of amoebic pathogenic mechanisms and may inform future therapeutic strategies.

## MATERIALS AND METHODS

### Amoebic strains and cell cultures

Trophozoites of *E. histolytica* HM-1:IMSS were grown axenically at 36.5°C in BI-S-33 medium containing 10% adult bovine serum (Sigma-Aldrich, St. Louis, MO, USA). Human intestinal epithelial cells (Caco-2) were grown in Eagle’s minimum essential medium (MEM) (#10-010-CV; Corning, Manassas, VA, USA) supplemented with 20% fetal bovine serum (HyClone Laboratories, Logan, UT, USA), MEM non-essential amino acids (Gibco, Grand Island, NY, USA), 2 mM L-glutamine (Gibco, Grand Island, NY, USA), and 1 mM sodium pyruvate (Gibco, Grand Island, NY, USA) at 37°C in a 5% CO_2_ incubator (31). Human embryonic kidney (HEK) 293 cells were cultured in OPM-293 CD05 medium (81075-001; Opmbiosciences, Shanghai, China) at 37°C, 5% CO_2_, and 120 rpm shaking.

### Expression and purification of eukaryotic Igl

Eukaryotic Igl was expressed and purified as previously described (8). The full-length *Igl1* gene (GenBank: AF337950.1) was amplified via PCR and subcloned into a pCMV6 plasmid (PS100001; OriGene) by double digestion with the restriction enzymes Sgf I and Mlu I (Fig. S3A). The primer sequences were as follows: forward, CCGCGATCGCATGGATTATACTGCTGATAAGCTC; reverse, CGACGCGTGAACATAAATGCTAACATGACTATCA. The pCMV6-*Igl* plasmid was amplified by *Escherichia coli* DH5α competent cells, then purified using the EndoFree Plasmid Maxi Kit (Qiagen, Hilden, Germany) (Fig. S3B). Plasmids were transiently transfected into HEK 293 cells using polyethyleneimine for protein expression. After 48 h of shaking, cells were harvested and lysed using 25 mM Tris-HCl (pH 7.4), 150 mM NaCl, 1% (vol/vol) NP-40, and 1 mM EDTA. Eukaryotic Igl was purified via anti-FLAG tag affinity chromatography and eluted with 0.1 M glycine (pH 2.75). The purity and immunoreactivity of the product were confirmed using 10% SDS-PAGE and western blotting (Fig. S3C and D). Hamster serum from amoebic liver abscess, hamster serum from Igl immunization, murine monoclonal antibody EH3015, and murine monoclonal antibody EH3077 were used as primary antibodies (1:100 dilution), while horseradish peroxidase-conjugated goat anti-mouse IgG and goat anti-hamster IgG (Abcam, Cambridge, United Kingdom) were used as secondary antibodies (1:500 dilution) (7). Protein bands were detected with an enhanced chemiluminescence Western Blotting Substrate Kit (Tanon, Shanghai, China).

### Sample treatment and preparation

For targeted metabolomics and metabolic flux analysis, Caco-2 cells were first seeded in six-well culture plates with a density of 5 × 10^5^ cells per well. In MEM (targeted metabolomics) or Dulbecco's modified Eagle medium containing 4.5 mg/mL [^13^C_6_]-glucose (metabolic flux analysis), 5 µg/mL eukaryote-expressed Igl was added for 12 h or 24 h stimulation. The cell culture supernatant was transferred for later use, while 500 µL extraction solution and 44 µL termination solution were successively added to each well. Due to the rapid metabolic rate of metabolites in processes such as glycolysis and tricarboxylic acid cycle, PBS washing was skipped and direct extraction was performed. Samples were stored at −80℃, then transferred to 4℃ and centrifuged at 15,000 rpm for 10 min before mass spectrometry. For single-cell transcriptomics, Caco-2 cells were cultured in T25 culture flasks. When the cell density reached 80%, 5 µg/mL eukaryote-expressed Igl was added for 24 h stimulation. After digestion and washing, 30 µm filter SmartStrainers (Miltenyi Biotec, Auburn, CA, USA) were used for filtration to obtain a single-cell suspension. The cell concentration was adjusted to 1,000 cells/µL.

### Targeted metabolomics and metabolic flux analysis

Chromatography separation was performed using the AB SCIEX ExionLC AD system equipped with an iHILIC-(P) Classic HPLC HILIC column (150 × 2.1 mm, 5 µm). Mobile phase A consisted of 95% water, 5% acetonitrile, 20 mM ammonium acetate, and 10 mM ammonia (50 mM methylene diphosphonic acid was added for non-targeted metabolic flux detections), while organic phase B consisted of 100% acetonitrile. The auto-sampler temperature was 6°C, the column temperature was 30°C, the flow rate was 0.2 mL/min, and the sample load was 5 µL. Liquid phase gradients were set as follows: 0 min, 85% B; 2 min, 85% B; 7 min, 60% B; 12 min, 35% B; 12.1 min, 20% B; 15.9 min, 20% B; 16 min, 85% B; 23 min, 85% B. Mass spectrometry of targeted metabolomics or metabolic flux analysis was performed using the AB SCIEX QTRAP 6500+ system (MRM scanning mode) or the TripleTOF 6600+ system (TOFMS+ production scanning mode), respectively. Positive and negative modes were separately scanned. Electrospray ionization source conditions were set as follows: ion source gas 1 as 60 psi, ion source gas 2 as 60 psi, curtain gas as 35 psi, source temperature as 500°C, ion spray voltage as 5,500 V or −4,500 V in positive or negative modes. Finally, ProteoWizard MSConvert was used to convert the original data format, EI-MAVEN was used for peak extraction to obtain the response information of each metabolite, and R version 4.3.1 was used for data analysis.

### Single-cell RNA-seq library preparation and sequencing

Caco-2 cells were loaded onto a 10X Genomics Chromium iX instrument, then single-cell libraries were prepared using a Single Cell 3′ library and Gel Bead Kit v3.1 in accordance with the manufacturer’s protocols. Generation of gel beads in emulsion (GEMs), barcoding, GEM-RT clean-up, complementary DNA (cDNA) amplification, and library construction were successively performed. After assessing the cDNA quality with a Qubit dsDNA HS Assay Kit (Invitrogen, Carlsbad, CA, USA), the final library pool was sequenced on an Illumina Novaseq 6000 platform using 150 bp paired-end reads.

### Single-cell RNA-seq data processing and analysis

Sample demultiplexing, barcode processing, alignment to the human genome (GRCh38), and raw gene expression counting were performed using Cell Ranger version 7.1.0 pipeline. Using R version 4.3.1, raw single-cell expression matrices were analyzed by the Seurat package version 4.3.0.1, while ambient RNA was removed by the SoupX package version 1.6.1. The criteria for the removal of low-quality cells were set as follows: (i) >10% unique molecular identifiers (UMIs) derived from the mitochondrial genome, (ii) <200 genes or >10,000 genes, and (iii) <500 UMIs. Doublets were identified by the DoubletFinder package version 2.0.3 with default settings, leaving 13,848 cells (both control and Igl-stimulated groups) and 24,520 genes for further analysis. After gene expression normalization using SCTransform version 2.0.3.5, single-cell RNA-seq data matrices from the two samples were integrated using the FindIntegrationAnchors and IntegrateData functions to remove the batch effect. Subsequently, linear dimensional reduction was performed with the RunPCA function, nonlinear dimensional reduction was performed with the RunUMAP or RunTSNE function, and cluster analysis was performed with the FindNeighbors and FindClusters functions.

For cell type annotation and cluster marker identification, the Shared Nearest Neighbor clustering algorithm from Seurat was used, with a resolution of 0.9. Cell types were assigned by examining the expression of marker genes and the top differentially expressed genes in each cluster. Cell clusters were visualized by the *t*-distributed stochastic neighbor embedding clustering algorithm. Signature genes of each cell cluster were identified by the FindAllMarkers function from Seurat. Percentages of different cell types were calculated accordingly.

### Differentially expressed genes identification and functional enrichment

DEGs were identified by the FindMarkers function from Seurat with the following parameters: min.pct  =  0.1; test.use = bimod. Genes with average log2 fold change (log2FC) value > 0.25 and adjusted *P* value < 0.05 were identified as DEGs, while genes with average log2FC value > 0.5 and adjusted *P* value < 0.05 were identified as significant DEGs. Gene Ontology, KEGG, Disease Ontology, and Reactome and Molecular Signatures Database enrichment analyses were conducted using the ClusterProfiler package version 4.8.2. Over representation analysis and gene set enrichment analysis were also conducted on DEGs. In the bioinformatic section, R version 4.3.1 was used for all statistical analyses, with a *P* value < 0.05 or an adjusted *P* value < 0.05 being considered statistically significant.

## Data Availability

Raw single-cell RNA-seq data are available through NCBI accession PRJNA1297965. Raw targeted metabolomics and metabolicflux data are available through the GitHub repository (https://github.com/MengFeng-Fudan/Data-Spectrum-ZYQ-2025).

## SUPPLEMENTAL MATERIAL

Supplemental materials spectrum.00381-25-s0001.pdf Supplemental methods, Table S1 and S2, and Fig. S1 to S3.

ASM does not own the copyrights to Supplemental Material that may be linked to, or accessed through, an article. The authors have granted ASM a non-exclusive, world-wide license to publish the Supplemental Material files. Please contact the corresponding author directly for reuse.
