# Supplementary material for: Multi-omics insights into the metabolic reprogramming of host cells triggered by Entamoeba histolytica Gal/GalNAc lectin intermediate subunit
Source: Microbiol Spectr. 2025 Aug 20;13(10):e00381-25. doi: 10.1128/spectrum.00381-25 (PMC12502685; doi:10.1128/spectrum.00381-25)
Supplement: Supplemental materials — Supplemental methods, Table S1 and S2, and Fig. S1 to S3. [file spectrum.00381-25-s0001.pdf]

SUPPLEMENTAL METHODS

Quantitative detection of intracellular metabolites

Caco-2 cells were first seeded in 96-well culture plates with a density of 1×10^4 cells per well, then stimulated with 5 μg/mL eukaryote-expressed Igl for 24 h. For glucose uptake and glutamine uptake detection, a Glucose Assay Kit-WST (G264; Dojindo, Kumamoto, Japan) and a Glutamine Assay Kit-WST (G268; Dojindo, Kumamoto, Japan) were respectively used. According to the manufacturer's instructions, optical density at 450 nm was measured, and concentrations were calculated using standard sample curves. For the detection of lactate production and cellular ATP amount under oligomycin or 2-deoxyglucose inhibition, a Glycolysis/OXPHOS Assay Kit (G270; Dojindo, Kumamoto, Japan) was used. An Oxygen Consumption Rate (OCR) Fluorometric Assay Kit (E-BC-F068; Elabscience, Wuhan, China) was applied for oxygen consumption rate measurement of Igl-treated Caco-2 cells. All experiments were performed at least thrice.

Western blotting of host intestinal epithelial cells

In addition to the immunogenicity detection of the eukaryotic expression of Igl, western blotting was conducted to confirm the effects of Igl on energy metabolism and autophagy in host intestinal epithelial cells. Caco-2 cells were first seeded in 6-well culture plates with a density of 5×10^5 cells per well, then stimulated with 5 μg/mL eukaryote-expressed Igl for 12 or 24 h. To verify the role of AMPK in the induction of host cell autophagy by *E. histolytica* Igl, Lipofectamine 2000 Transfection Reagent (Invitrogen, Carlsbad, CA, USA) was used for siRNA transfection to silence the *PRKAA1* gene (NCBI Gene ID 5562). The primer sequences used were as follows: *control forward*-UUCUCCGAACGUGUCACGUTT; *control reverse*-ACGUGAC ACGUUCGGAGAATT; *PRKAA1 forward*-GCAGAAGUAUGUAGAGCAAUCTT; *PRKAA1 reverse*-GAUUGCUCUACAUACUUCUGCTT. Transfection experiments were conducted according to the manufacturer's instructions, and cells were incubated with siRNA at a final concentration of 50 nM in antibiotic-free MEM; the medium was

changed 6 h after transfection. Similarly, to verify the role of mTOR in Igl stimulation, rapamycin (HY-10219; MedChemExpress, NJ, USA) was used for mTOR inhibition to promote host cell autophagy, and Caco-2 cells were treated with 20 nM rapamycin for 1 h before medium change and Igl addition. Each experiment was performed at least thrice.

Protein samples from Caco-2 cells were treated as described previously (1). Briefly, after being separated on 10% polyacrylamide gels and electrotransferred onto polyvinylidene difluoride membranes (General Electric Co., Schenectady, NY, USA), membranes were blocked with 5% BSA in PBS, then incubated with the following primary antibodies: anti-β-actin antibody (#3700; Cell Signaling Technology, Boston, MA, USA), anti-AMPKα antibody (#5831; Cell Signaling Technology), anti-phospho-AMPKα antibody (#2535; Cell Signaling Technology), anti-AKT antibody (#4691; Cell Signaling Technology), anti-phospho-AKT antibody (#9271; Cell Signaling Technology), anti-ULK1 antibody (#8054; Cell Signaling Technology), anti-mTOR antibody (#2983; Cell Signaling Technology), anti-raptor antibody (#2280; Cell Signaling Technology), anti-LC3A/B antibody (#12741; Cell Signaling Technology), and anti-SQSTM1/p62 antibody (#88588; Cell Signaling Technology). The secondary antibodies were HRP-conjugated goat anti-rabbit IgG H&L (Ab6721; Abcam, Cambridge, United Kingdom) and HRP-conjugated goat anti-mouse IgG H&L (Ab6789; Abcam). Proteins were detected with an ECL Western Blotting Substrate Kit (Tanon, Shanghai, China).

MDC fluorescent probe staining for autophagy detection

Using MDC as a fluorescent probe, laser confocal microscopy (SP8; Leica Microsystems, Wetzlar, Germany) and microplate reader quantification (Agilent BioTek, Vermont, USA) were performed to detect Caco-2 cell autophagy levels after Igl stimulation. First, Caco-2 cells were separately seeded in 20-mm diameter glass bottom sterile Petri dishes with a density of 3×10^5 cells per well (for the confocal microscope) and 96-well culture plates with a density of 2×10^4 cells per well (for the microplate reader), then stimulated with 5 μg/mL eukaryote-expressed Igl for 12 or 24

h. An Autophagy Staining Assay Kit with MDC (Beyotime, Shanghai, China) was used for detection. The medium was aspirated, and 100 μL of 1X MDC dye solution was added to each well, followed by a 30-min incubation at 37°C. Afterward, the MDC dye solution was removed, and the wells were washed thrice with 1X Assay Buffer. Finally, 100 μL of 1X Assay Buffer was added to each well. Fluorescence intensities were measured using a laser confocal microscope (350 nm excitation) and a Synergy H1 Microplate Reader (335 nm excitation; 512 nm emission). All experiments were performed at least thrice.

References

1. Zhang H, Jin K, Xiong K, Jing W, Pang Z, Feng M, Cheng X. 2023. Disease-associated gut microbiome and critical metabolomic alterations in patients with colorectal cancer. Cancer Med 12:15720-15735.

Supplementary Table 1: Differentially expressed mTOR and autophagy genes in the Igl-treated group

Gene Symbol	log2FC	p_val
SNX5	0.44210322	2.64E-286
EEF1A1	0.23121007	8.19E-168
RICTOR	0.202853887	9.68E-71
CSNK2A1	0.184357742	3.11E-72
VPS41	0.167176066	5.23E-64
SQSTM1	0.164684816	1.54E-38
ATG12	0.160178202	2.13E-60
CSNK2A2	0.160003917	3.24E-54
TOMM20	0.14884238	1.15E-57
LARS	0.133532687	2.62E-36
MTDH	0.126257061	1.66E-30
EXOC4	0.119174019	1.15E-32
GOLGA2	0.105682389	8.77E-22
VTI1A	0.08949201	1.43E-26
VPS36	0.067074407	1.45E-21
HK2	0.066425569	8.72E-28
MCL1	0.0527816	3.93E-06
TOMM40	0.051734831	7.48E-23
MFN2	0.047770473	3.35E-08
ULK1	0.039398446	1.20E-09
RAB5A	0.034108979	2.36E-10
VTA1	0.029326037	1.04E-03
LPIN2	0.025698705	8.86E-25
SLC3A2	0.025289966	6.07E-10
TOMM22	0.022521833	7.04E-09
PGAM5	0.017107919	3.02E-02
SRC	0.014294395	4.26E-02
ATG16L1	0.01388264	8.38E-06
PIK3C3	0.013178642	1.16E-02
LAMTOR5-AS1	0.012718924	1.72E-01
LAMTOR5-AS1	0.012718924	1.72E-01
SEH1L	0.008648864	4.96E-28
TOLLIP	0.007649955	1.91E-02
CSNK2B	-0.004963031	1.57E-25
CSNK2B	-0.004963031	1.57E-25
MFN1	-0.010901762	6.44E-02
MFN1	-0.010901762	6.44E-02
SMURF1	-0.013001553	5.65E-01
ARSB	-0.016919097	3.41E-02
TM9SF1	-0.017967301	1.94E-01

PINK1	-0.020738146	2.83E-04
TOMM7	-0.025166636	3.67E-03
FUNDC1	-0.03584836	7.73E-03
ATG5	-0.063009663	1.09E-05
HTRA2	-0.076848335	2.33E-13
PIK3R1	-0.084776334	1.09E-11
EIF4E2	-0.097376802	8.93E-43
MVB12A	-0.099967369	2.25E-17
MAP1LC3B	-0.141557449	3.61E-34
LAMTOR2	-0.182055263	1.02E-69
LAMTOR2	-0.182055263	1.02E-69
VDAC1	-0.210972843	1.87E-76
RPS27A	-0.220869124	2.15E-198
UBC	-0.236227724	6.10E-75
ATP6V1G1	-0.23758974	4.02E-94
ATP6V1G1	-0.23758974	4.02E-94
UBA52	-0.239596301	7.52E-181
GABARAPL2	-0.301390124	5.15E-145
TOMM5	-0.313828085	1.30E-125
ATP5IF1	-0.319924901	4.23E-150
UBB	-0.327479757	3.18E-183
TOMM6	-0.478427993	1.01E-321

Supplementary Table 2: The metabolites included in Warburg effect, glycolysis, and gluconeogenesis pathway.

**Warburg effect**	**Glycolysis**	**Gluconeogenesis**
Glucose	Glucose	Lactate
Glucose-6-phosphate	Glucose-6-phosphate	Glycerol
Fructose-6-phosphate	Fructose-6-phosphate	Glucogenic amino acids
Fructose-1,6-bisphosphate	Fructose-1,6-bisphosphate	Pyruvate
Glyceraldehyde-3-phosphate	Glyceraldehyde-3-phosphate	Oxaloacetate
1,3-Bisphosphoglycerate	1,3-Bisphosphoglycerate	OAA
3-Phosphoglycerate	3-Phosphoglycerate	Phosphoenolpyruvate
Phosphoenolpyruvate	2-Phosphoglycerate	Malate
Pyruvate	Phosphoenolpyruvate	Aspartate
Lactate	Pyruvate	Glucose-6-phosphate
NAD^+/NADH	ATP	Fructose-6-phosphate
Acetyl-CoA		Fructose-1,6-bisphosphate
Citrate		Glyceraldehyde-3-phosphate
α-Ketoglutarate		3-Phosphoglycerate
Succinate		2-Phosphoglycerate
Fumarate		ATP
Malate		GTP
6-Phosphogluconate		
Ribulose-5-phosphate		
ATP		
ADP		
AMP		

Supplementary Figure Legends

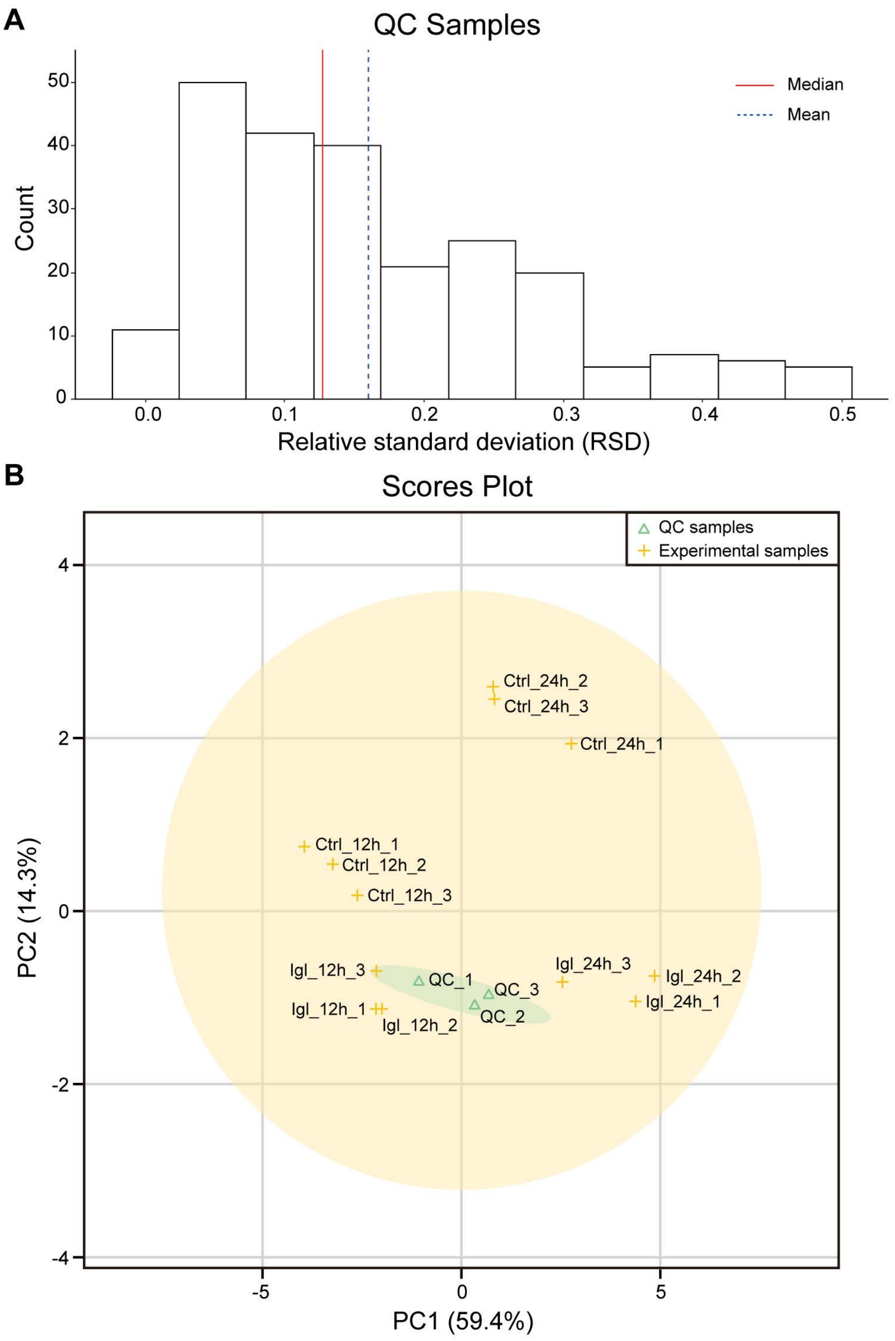

C

Internal Standard

Name	Mean	SD	RSD
4,4,5,5-tetradeuterolysine	20384648	1233005	0.06

Fig. S1 Quality control of targeted metabolomics data. (A) Relative standard deviation based on quality control samples. (B) Principal component analysis of quality control samples and experimental samples. (C) Stable isotope-labelled internal standard.

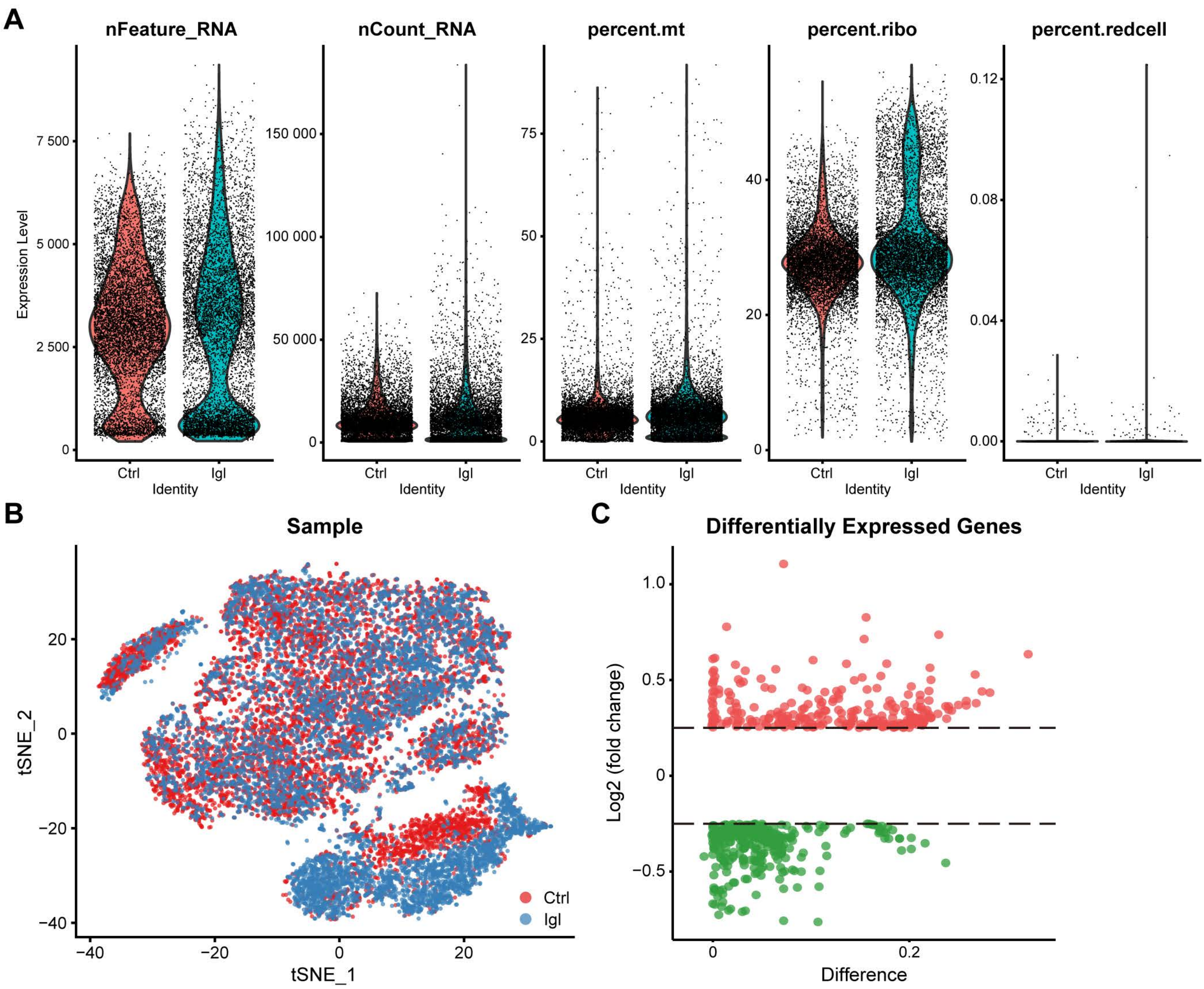

Fig. S2 Quality control of single-cell RNA-seq data. (A) For individual cells in the transcriptomic data, number of total expressed genes (nFeature_RNA), number of transcripts (nCount_RNA), percentage of mitochondria genes (percent.mt), percentage of ribosomal genes (percent.ribo), and percentage of red blood cell genes (percent.redcell) were analyzed to filter low-quality information after single-cell sequencing, such as fragmentary cells, double cells, and dead cells. (B) Cell t-SNE clustering analysis of integrated samples from control and Igl-treated cells. (C) Effects of Igl stimulation on the transcriptional profile of Caco-2 cells. Volcano plot showing significantly differentially expressed genes in Caco-2 cells after Igl treatment.

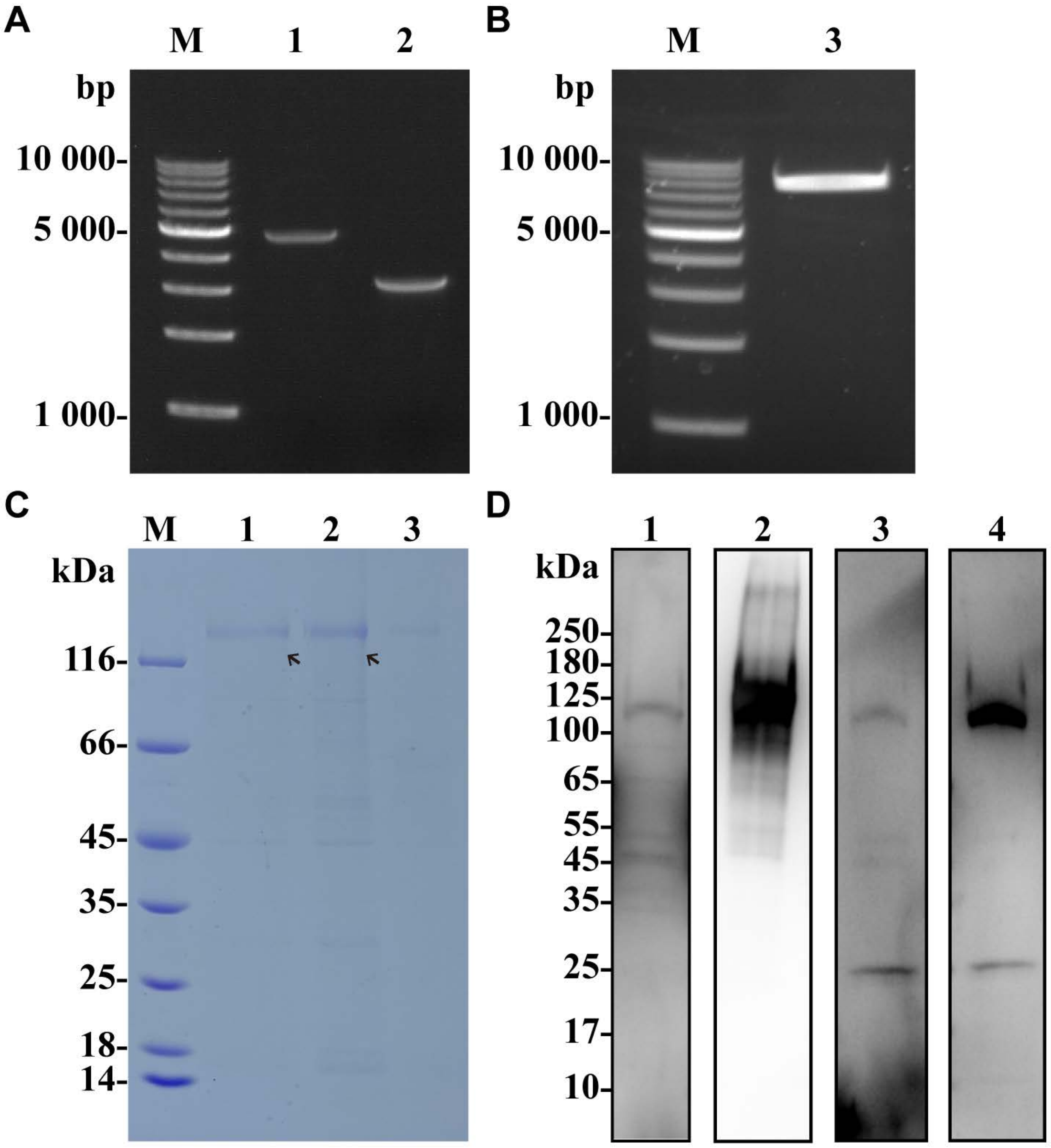

Fig. S3 Eukaryotic expression, purification, and immunoreactivity identification of Igl protein. (A and B) The pCMV6-Igl plasmid constructed for recombinant Igl expression. Agarose gel electrophoreses of pCMV6 empty vector (lane 1,4919 bp), Igl1 gene (lane 2,3264 bp), and the pCMV6-Igl plasmid after digestion (lane 3,8147 bp) were shown. M, Takara 1 kb DNA Marker. (C) SDS-PAGE analysis of purified recombinant Igl protein. Protein bands were visualized with Coomassie Brilliant Blue. Lane 1-3, elution fractions with arrows pointing to the protein bands of Igl. M, standard protein marker. (D) Identification of the immunoreactivity of recombinant Igl protein by western blotting. Primary antibodies: hamster serum with amoebic liver abscess (lane 1), hamster serum of Igl immunization (lane 2), murine monoclonal antibody EH3015 (lane 3), and murine monoclonal antibody EH3077 (lane 4). Secondary antibodies: HRP-conjugated goat anti-mouse IgG and goat anti-hamster IgG.
